# Perineural invasion as a predictor of local recurrence in cats with squamous cell carcinoma treated with electrochemotherapy

**DOI:** 10.3389/fvets.2024.1408260

**Published:** 2024-06-06

**Authors:** Francielle Fernanda Quirino dos Santos, Laís Calazans Menescal Linhares, Michelle do Carmo Pereira Rocha, Krishna Duro de Oliveira, Marcelo Monte Mor Rangel, Andrigo Barboza de Nardi

**Affiliations:** ^1^School of Agricultural and Veterinary Sciences, Sãoo Paulo State University (UNESP) “Júlio de Mesquita Filho”, Jaboticabal, Brazil; ^2^VCLab Pathology, São Paulo, Brazil; ^3^Vet Cancer Veterinary Oncology, São Paulo, Brazil

**Keywords:** feline, oncology, skin cancer, prognostic factors, nervous sheath

## Abstract

**Introduction:**

Squamous cell carcinoma (SCC) is a malignant neoplasm that accounts for approximately 15–25% and 70–80% of all feline cutaneous and oral tumors, respectively. Similar to that in humans, feline SCC can be highly invasive locally; however, its metastasis rate is low. Thus, effective local treatment may be curative for most patients, and includes surgery, electrochemotherapy (ECT), cryosurgery, or a combination of these. However, this neoplasia can manifest more aggressively in some patients, leading to higher recurrence rates. In humans, perineural invasion (PNI) has been described as a relevant tumor dissemination pathway associated with high-risk SCC, resulting in higher recurrence rates, resistance to local treatments, and short survival. However, PNI and its prognostic value have not been described in feline SCC. This study aimed to evaluate the PNI in a feline population with SCC treated with ECT and correlate its presence with the occurrence of local recurrence and other clinical variables.

**Methods:**

Twenty-four cats histopathologically diagnosed with SCC between 2013 and 2021 were retrospectively selected from the medical records of the Oncological Center Vet Cancer (São Paulo, SP, Brazil). The inclusion criteria were ECT as the sole therapy, histopathological evaluation of PNI, and absence of distant metastatic disease.

**Results:**

The complete response rate was 96% (23/24), and PNI was identified in 33% of the cats (8/24, PNI-positive group), whereas 67% were free of this invasion (16/24, PNI-negative group). All PNI-positive cats developed local recurrence, whereas only five PNI-negative cats experienced recurrence. Local recurrence was significantly associated with PNI (*p* = 0.03).

**Discussion:**

The results of this study are preliminary but promising. The data obtained are the first regarding PNI occurrence in feline SCC and pave the way for further studies, mainly to correlate the PNI with survival data and better define its prognostic value.

## 1 Introduction

Squamous cell carcinoma (SCC) is a malignant neoplasm arising from the squamous epithelium and is mainly found in the skin, oral cavity, esophagus, nail beds, and footpads. SCC is one of the three most common tumors of feline skin, representing approximately 15–25% of all skin neoplasms. SCC is the most common oral cancer in cats (1–3).

The etiology of cutaneous SCC is closely associated with chronic exposure to UV radiation, and cats with white and glabrous coats are highly predisposed to its development (4). The most commonly affected anatomical sites include sparsely haired and non-pigmented regions of the head, such as the pinna of the ear, temples, the nasal planum, and the periocular region. These locations are at great risk due to poor fur coverage and high exposure to sunlight (1, 2).

Initially, SCC lesions can be superficial and non-invasive; however, they can evolve into invasive SCC in some cases, presenting infiltrative behavior and destroying tumor-adjacent structures, such as bone and cartilage. The metastasis rate is usually low, and its development is relatively slow, mainly affecting the lymph nodes and lungs (1).

The main factors that influence therapeutic decision-making are tumor location and staging, which include the size and extent of the lesion (5). Given the low propensity scores for metastatic dissemination, local therapy, including surgery, radiotherapy, cryotherapy, and electrochemotherapy, is the mainstay of treatment for cutaneous SCC (2, 6–8). Surgery is the gold-standard method of treatment and offers the highest local cure rates. However, obtaining safety margins is difficult in some cases because of the complex location of the lesions and advanced stage (T3 and T4) at diagnosis (2, 8).

Similar to cats, SCC is a malignant cutaneous neoplasm that affects humans worldwide and is also considered a locally invasive neoplasm, which can become difficult to control surgically locally due to late diagnosis and advanced stage. In addition, feline and human SCC share several similarities in biological behavior, pathogenesis, etiology factors, and response to therapy (9).

Electrochemotherapy (ECT) is a local therapeutic approach that combines systemic or intratumoral administration of low-permeability drugs with the application of an electric field. ECT has been proven effective against tumors of different histology, such as carcinomas, sarcomas, mast cell tumors, and melanomas. In human and veterinary medicine, ECT is a standardized treatment for SCC, resulting in objective responses of 83–96%, in humans and cats. ECT stands out for its efficacy and selectivity, which destroys tumor cells while preserving adjacent healthy tissue, ensuring treatment of the margins of the lesion. This effect provides greater chances for cosmetic, anatomical, and functional conservation in patients. However, similar to that with surgery, the results depend on the extent of the injury (8, 10–12).

Although most humans and cats with SCC can be successfully treated with current treatment options and have a good prognosis, some may develop the disease in its invasive, aggressive, and recurrent form, even with the implementation of appropriate therapies. For human SCC, different histopathological features were associated with worse prognosis and recognized as prognostic factors, allowing the recognition of “high-risk” patients (13–15). Among these features, perineural invasion (PNI) is considered an independent prognostic marker associated with higher rates of local recurrence and metastases, and a worse prognosis (14–17). The incidence of PNI in human cutaneous SCC ranges from 2 to 14%, increasing to 24% in recurrent cases and up to 70% of the oral SCC cases (15, 18).

Perineural invasion is a complex and reciprocal pathway of tumor dissemination that involves dynamic interactions between tumor cells and nervous components (such as Schwann cells and neurons), culminating in tumor migration, invasion, and proliferation around and within the nerves (19). In general, neurotropic factors, or neurotrophins, are released by tumor cells in the local microenvironment and are detected by Schwann cells and neurons, which, in response, also secrete neurotrophins that promote the proliferation, invasion, and migration of the tumor toward the nerves (19–21). Neuronal cell adhesion molecule-1 (NCAM-1), nerve growth factor (NGF), and glial cell lineage-derived neurotrophic factor (GDNF), are examples of neurotrophins involved in this process. The respective receptors for each neurotrophin are also expressed in tumors and nerve cells (19, 20).

However, few prognostic factors have been established for cats with SCC. Tumor stage is considered the main prognostic factor; however, advanced stage is a late marker that is mostly detected when few local control options remain (8, 22). To date, there is no evidence in the literature regarding the incidence of PNI in cats with SCC and its association with tumor behavior and prognosis. Given the similarity between human and feline SCC and the relevance of PNI in the pathogenesis and prognosis of human SCC, its investigation represents a starting point for identifying “high-risk” feline patients. This is essential for identifying patients with a greater chance of refractoriness to treatment and worse prognosis, providing better therapeutic planning.

Thus, the primary objective of the present study was to evaluate perineural invasion in feline SCC and to correlate its presence with the occurrence of local recurrence and other clinical variables in a population of felines with SCC treated with ECT.

## 2 Materials and methods

### 2.1 Ethical aspects

The present retrospective clinical study was conducted in accordance with the guidelines and regulations of the National Council for the Control of Animal Experimentation (CONCEA) and was approved by the Animal Experimentation Ethics Committee of the Universidade Estadual Paulista “Júlio de Mesquita Filho” (UNESP/FCAV), Jaboticabal/SP (protocol no. 9849/2023).

### 2.2 Patient selection

The medical records of client-owned cats treated with ECT for a confirmed diagnosis of SCC at the Vet Cancer Oncology Center (São Paulo, SP, Brazil) between January 2013 and December 2021 were retrospectively reviewed. The inclusion criteria were a histopathological diagnosis of SCC, histopathological evaluation of PNI, and use of ECT as the only treatment. The exclusion criteria were the presence of distant metastases at diagnosis and patients undergoing surgery or other local and systemic treatments associated with ECT.

Data collected from medical records included sex, age, breed, location and number of lesions, assessment of distant metastases (abdominal ultrasound and chest radiography), ECT protocol used, number of ECT sessions, adverse effects, initial response to ECT, and development of local recurrence. Information on tumor size, exact relapse moment, treatment performed after relapse, disease-free time, and survival time was not available for all cases and was therefore not included in this study.

All histopathological reports were reviewed to collect data regarding the diagnosis and presence of PNI. The cats were divided into two groups: PNI-positive and -negative groups for patients in which PNI was present and absent, respectively.

In all cases, a definitive diagnosis was obtained through intraoperative histopathological analysis, and ECT was immediately performed. All procedures were performed under general anesthesia. For anesthetic induction, propofol (Lipuro® 10 mg/mL; Braun, Melsungen, Germany) was used intravenously, at a dose of 5 mg/kg, followed by endotracheal intubation. Isoflurane (Generic, Laboratório Biochimico, Itatiaia, Rio de Janeiro, Brazil) was used to maintain anesthesia.

### 2.3 Intraoperative histopathology

**Figure 1:**
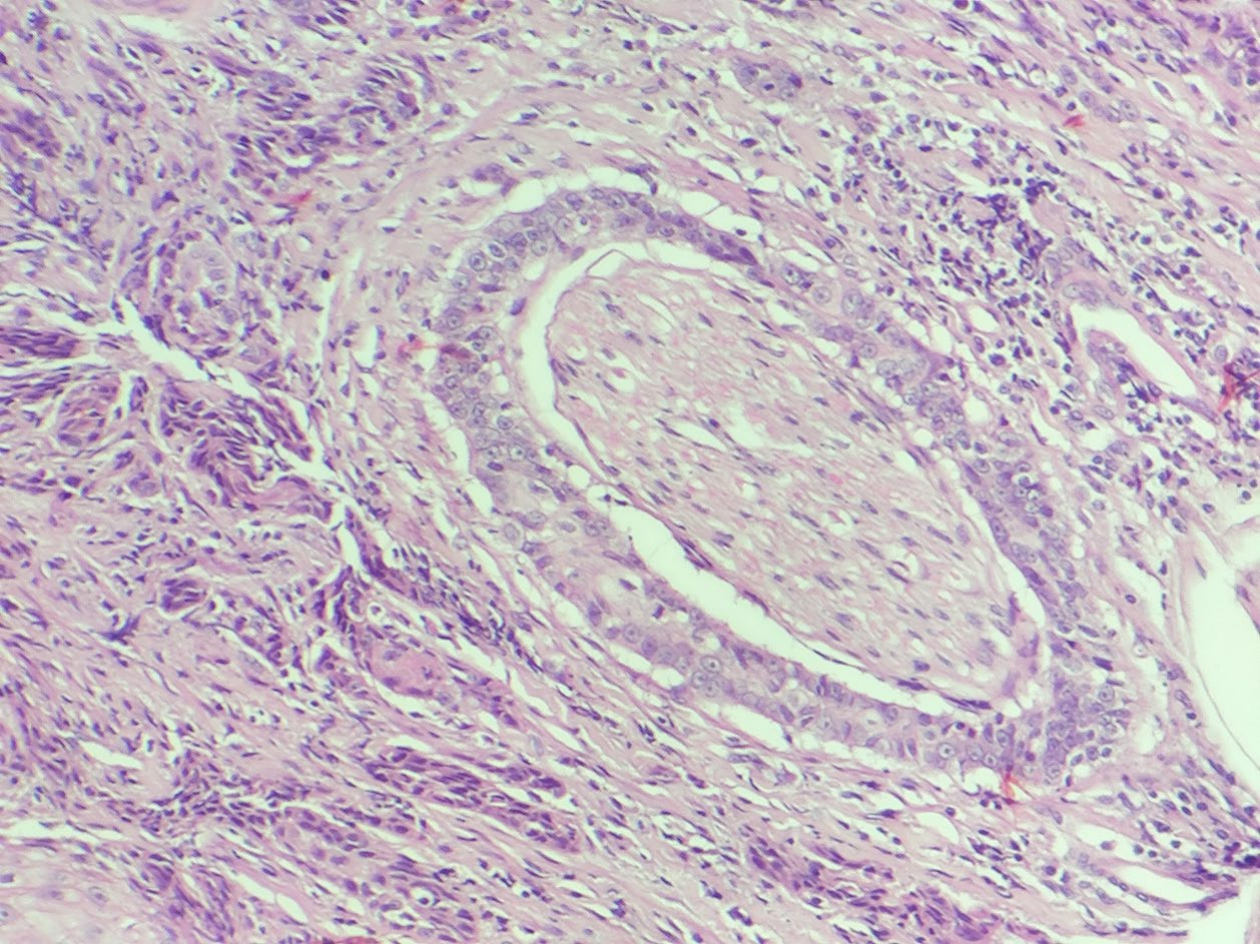
Histological photomichograph of feline SCC (PNI-positive), presenting tumoral cells completely surrounding the nerve and inside it (H&E).

For the intraoperative histopathological evaluation, freezing histopathology was employed, comprising the steps of macroscopic analysis of the sample, cleavage, freezing with compressed CO_2_, and preparing approximately 5 μm thick sections, on a portable freezing microtome (Leica). The sections were stained with toluidine blue and analyzed under a light microscope (Nikon YS2). The tumor samples were stored in a 10% formaldehyde solution for conventional processing and subsequent analysis on histological slides stained with hematoxylin and eosin for confirmation and a more detailed description of the morphological characteristics of the neoplasm, including the assessment of PNI (Figure 1). The sample was considered PNI-positive when tumor cells are invading the nerve and the evaluation of PNI considered the standard area of 2.37 mm^2^ and the number of fields varied between 12 and 15. All the histopathogical evaluations (frozen section and conventional) were made by the same broad certified pathologist.

### 2.4 Electroporation protocol

Electrochemotherapy was initiated with the intravenous administration of bleomycin (Bleovet®), at a dose of 15,000 IU/m^2^, diluted in 5 mL of saline solution. After 8 min, electrical pulses were applied to the entire length of the tumors as well as to the surrounding healthy margins. The application was performed within 40 min (12).

The protocol established consisted of the application of eight high-voltage electrical pulses, with a voltage of 1,300 V/cm, a frequency of 5 KHz, and a duration of 100 μs, for each shot performed. The pulse generating device used was the VETCP 125® (VET CÂNCER/IMPLASTIC, São Paulo, SP, Brazil), which is composed of two sequences of three stainless steel needles (1.1 mm thick each), equidistant by 5 mm with 3 mm between the ipsilateral needles.

After completing the procedure, the cats were administered dipyrone (25 mg/kg, IV, single dose) or ketoprofen (1 mg/kg, PO, single dose). In the absence of complications, the patient was discharged with home prescriptions of dipyrone (25 mg/kg, orally, SID, 3–5 days), ketoprofen (1 mg/kg, orally, SID, 3–5 days), and amoxicillin with potassium clavulanate (15 mg/kg, PO, BID, 7 days).

The cats were examined on days 7, 14, 21, and 30 after the procedure and every 30 days thereafter. At each return visit, a general clinical examination of the patient was performed, as well as a complete blood count and biochemical tests (creatinine, urea, ALT, and FA). Abdominal ultrasonography and thoracic radiography were performed every 3 months, for staging purposes or according to the animal’s needs.

### 2.5 Response evaluation

According to the RECIST response evaluation criteria (23), local responses were categorized as complete response (CR), partial response (PR), stable disease (SD), or progressive disease (PD). A response assessment was performed 30 days after each ECT session, and a new session was recommended if there was evidence of macroscopic disease. In cases where small areas suspicious for neoplastic lesions were identified, a fresh incisional biopsy was performed to confirm and plan an additional ECT session.

### 2.6 Safety evaluation

Adverse effects were evaluated, and local toxicity was classified using a six-point toxicity score developed by Lowe et al. (24): 0, no toxicity; 1, mild edema; 2, moderate edema/necrosis <1 cm; 3, severe edema; 4, deep necrosis; and 5, severe edema and tissue loss.

### 2.7 Statistical analyses

Perineural invasion presence, recurrence rate, and toxicity score were previously subjected to the Shapiro–Wilk normality test, and if there was no normal distribution, the Wilcoxon test was used to compare the variables. Furthermore, the groups with and without perineural sheath involvement were subjected to principal component analysis (PCA), according to PNI presence and recurrence rate. Statistical calculations were performed using the R® software program (version 4.2.2, 2022), with a significance level of 95% (*p* < 0.05).

## 3 Results

In this study, 24 felines were selected and 30 SCC lesions were diagnosed using histopathology. Of these cats, 96% (23/24) were domestic short hair cats (DSH), and only one (4%) was Angora. Other demographic data regarding the average age at diagnosis and the distribution of injuries according to sex are presented in Table 1.

**Table 1:** Demographic characteristics of the included cats.

Sex	*n*	%	Age (years)	Number of lesions
Male	9	37.5	11.4 ± 2.9	16
Female	15	62.5	10.4 ± 3.2	17
Total	24	100		33

**Figure 2:**
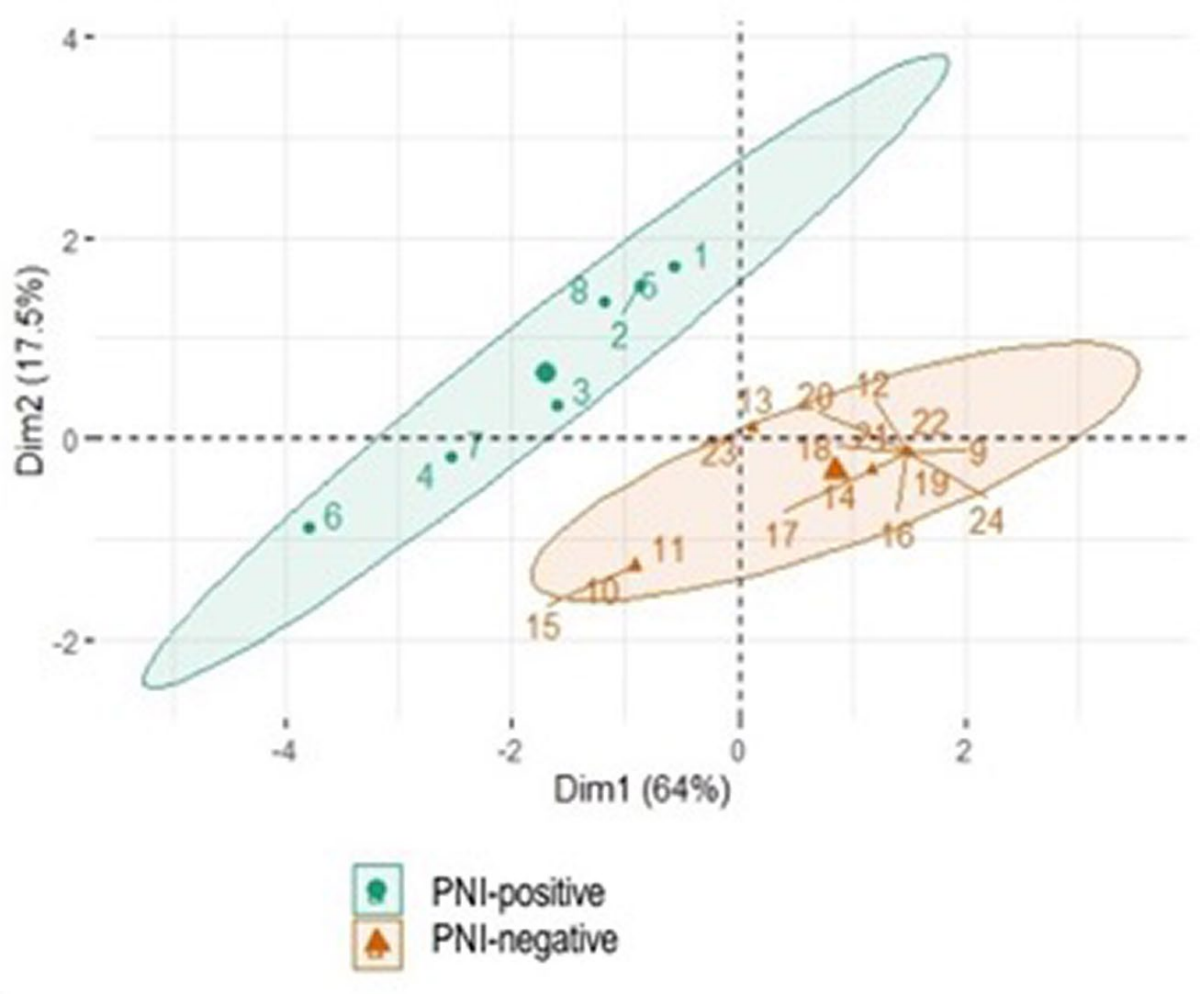
Significant difference between the groups using Principal Component Analysis (PCA). The variables considered were presence of PNI and recurrence rate.

A total of 33% of cats were PNI positive (8/24 IPN-positive), whereas 67% were PNI negative (16/24 IPN-negative) (Table 2). Considering the PCA, it was possible to verify grouping by similarity according to PNI presence (PNI-positive and –negative groups) and recurrence rate, confirming that there was a significant difference between them (Figure 2).

**Table 2:** General case information and treatment outcome.

Patient #	Tumor location	PNI	# of ECT sessions	Initial response	Local recurrence
1	Chin	Yes	1	CR	Yes
2	Lip	Yes	2	CR	Yes
3	Ear	Yes	1	CR	Yes
4	Nasal planum	Yes	4	CR	Yes
5	Ear	Yes	1	CR	Yes
6	Eye canthus	Yes	8	CR	Yes
7	Face (NS)	Yes	4	CR	Yes
8	Lip	Yes	3	CR	Yes
9	Nasal planum	No	1	CR	No
10	Nasal planum	No	2	CR	Yes
11	Chin	No	2	CR	Yes
12	Oral cavity	No	1	CR	No
13	Multiple sites^a^	No	2	CR	Yes
14	Ear	No	2	CR	No
15	Multiple sites^b^	No	2	CR	Yes
16	Nasal cavity	No	1	CR	No
17	Multiple sites^c^	No	1	PR	No
18	Ear	No	1	CR	No
19	Ear	No	1	CR	No
20	Nasal planum	No	1	CR	No
21	Nasal planum	No	1	CR	No
22	Eyelid	No	1	CR	No
23	Ear	No	1	CR	Yes
24	Ear	No	1	CR	No

^a^Nasal planum and chin region; ^b^Nasal planum, eye, nasal cavity, and lips; ^c^Nasal planum, temporal region, and eyelid. NS, Non-specified, ECT, Electrochemotherapy, PR, Partial response, and CR, Complete response. PNI, Perineural invasion.

All cats presented with SCC lesions on their head. Of the 24 cats, 20 developed a single lesion in one location on the head, and three developed multiple lesions (two or more) in different locations on the head. In general, the most affected locations were the nasal planum (33%; 8/24) and ear (29%; 7/24). The remaining regions included the oral cavity, lip, nasal cavity, eyelid, eye canthus, eye, and temporal and chin regions (Tables 2, 3). In the PNI-negative group, the most affected sites were the nasal planum (7/16, 44%) and ear (5/16, 31%). In the PNI-positive group, 50% of the affected sites were the ears and lips, with two patients each (Table 3).

**Table 3:** Distribution of the lesions according with anatomical location between PNI-positive and PNI-negative groups.

Tumor location	# of lesions (n)	Total (%)	PNI-positive (*n* = 8)	PNI-positive (%)	PNI-negative (*n* = 16)	PNI-negative (%)
Nasal planum	8	26.7	1	12.5	7	44
Ear	7	23.3	2	25	5	31
Lips	3	10	2	25	1	6.2
Oral cavity	1	3.3	0	0	1	6.2
Nasal cavity	2	6.7	0	0	2	12.5
Eyelid	2	6.7	0	0	2	12.5
Eye	1	3.3	0	0	1	6.2
Eye canthus	1	3.3	1	12.5	0	0
Temporal region	1	3.3	0	0	1	6.2
Chin	3	10	1	12.5	2	12.5
Non-specified	1	3.3	1	12.5	0	0
Total of lesions	30	100	8	-	22	-

**Figure 3:**
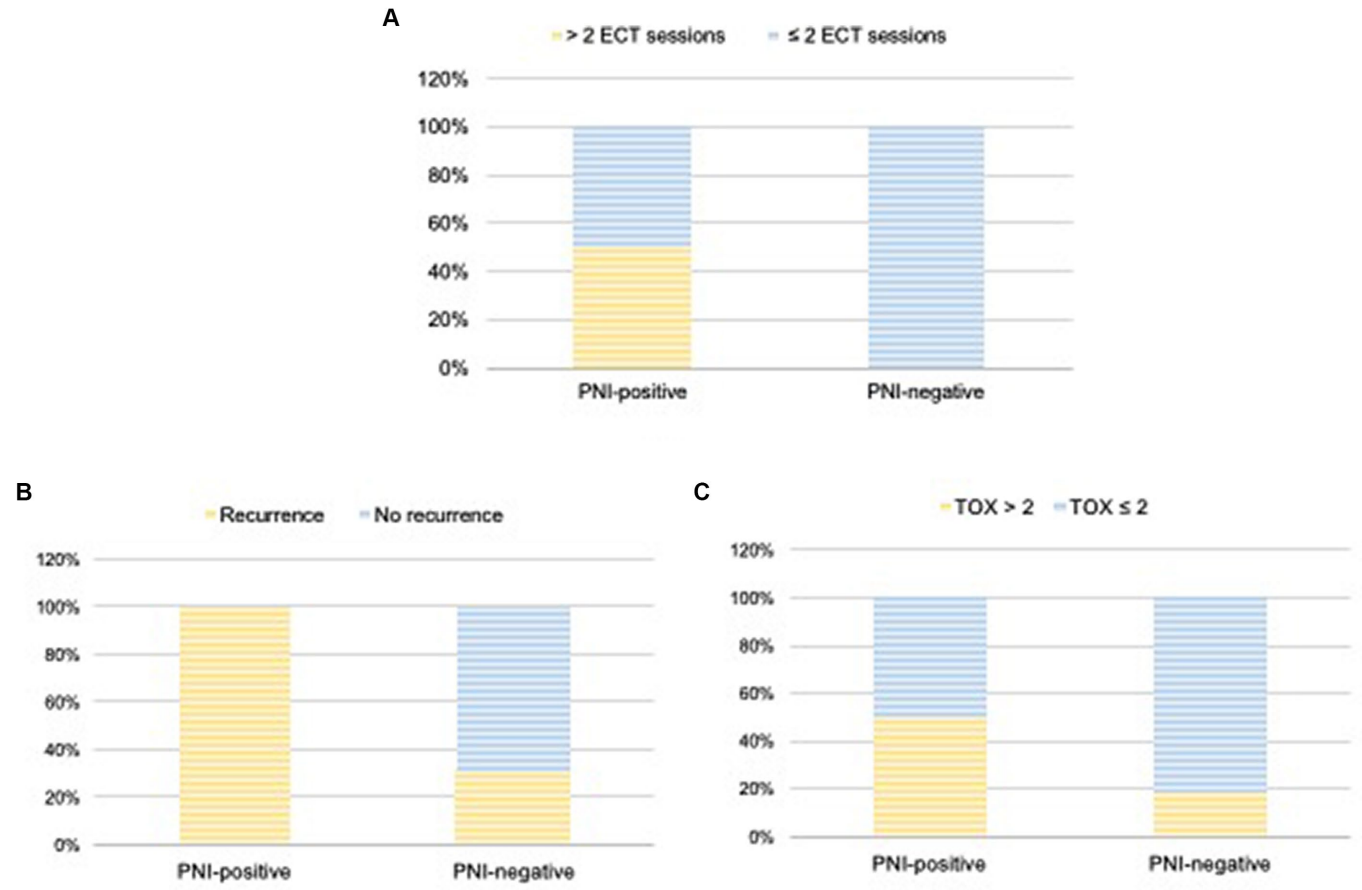
Number of ECT sessions **(A)**, local recurrence **(B)**, and TOX scores **(C)** according with the presence of PNI (PNI-positive × PNI-negative groups). Wilcoxon test was used to compare TOX scores and recurrence between the groups.

The number of ECT sessions performed varied from one to eight (Table 2). Of the 24 cats, 13 (54.2%) underwent one session; 7 (29.2%) underwent two sessions; 1 (4.2%) underwent three sessions, 2 (8.4%) required four sessions, and 1 (4.2%) required eight sessions. All PNI-negative cats underwent 1–2 ECT sessions (11 patients, one session; five patients, two sessions). In contrast, 50% of the PNI-positive cats underwent 1–2 sessions (three patients, one session and one patient, two sessions), whereas 50% underwent 3–8 sessions (one patient, three sessions; two patients, four sessions, and one patient, eight sessions) (Figure 3A).

Twenty-three cats (96%) achieved CR initially and only one achieved PR (PNI-negative), resulting in an initial overall response rate of 100%. Local recurrence occurred in 13 patients, with an overall recurrence rate of 54%. Five of the 13 cats that experienced local recurrence (38.4%) were PNI negative, with a recurrence rate of 31% (5/16). In contrast, all eight PNI-positive cats developed local recurrence (100% recurrence rate) (Figure 3B). Therefore, according to the Wilcoxon test, the local recurrence rate was significantly higher in cats with PNI than in those without PNI (*p* = 0.03).

No patient died because of the ECT or local toxicity caused by ECT. Overall, the local toxicity score (TOX) was ≤2 in 16.5% of patients (4/24) and > 2 in 67% (16/24). Adverse events were not detailed in the medical records for four of the 24 cats examined in this study, making it impossible to determine their TOX score (Table 4). Of the 16 cats with TOX > 2, five were PNI-positive (5/8; 62.5%) and 11 were PNI-negative (11/16; 68.7%) (Figure 3C). Among the 16 cats with TOX > 2, eight developed local recurrence and eight did not. According to the Wilcoxon test, no significant correlation was observed between TOX and the occurrence of PNI (*p* = 0.21) or recurrence (*p* = 0.42).

**Table 4:** TOX score classification between PNI-positive and PNI-negative groups.

TOX score^1^						
	1	2	3	4	5	NS
PNI-positive (*n*)	0	2	4	0	1	1
PNI-negative (*n*)	1	1	5	3	3	3
Total *n* (%)	1 (4%)	3 (12.5%)	9 (37.5%)	3 (12.5%)	4 (16.6%)	4 (16.6%)

^1^According to Lowe et al. (24). NS, Non-specified.

## 4 Discussion

The identification of high-risk human SCC is based on various factors, including the depth of invasion, tumor differentiation, and PNI. PNI has been identified as one of the most important markers of malignancy in humans with cutaneous and oral SCC and is associated with worse prognosis, high rates of local recurrence (16–45%), and lymph node metastasis (10–50%) (25).

Although the PNI is a recognized and standardized prognostic factor for human SCC, no studies have evaluated its occurrence or significance in feline SCC. Thus, this study aimed to retrospectively evaluate the occurrence of PNI in cats with SCC treated with ECT, and to correlate its presence with the occurrence of local recurrence and other clinical variables.

For standardization, all patients selected in this study received ECT as a single local treatment. Currently, ECT with bleomycin can be considered a first-line local treatment option for cats with SCC, standing out for its capacity to selectively target cancer cells while preserving healthy surrounding structures, and for its antitumor efficacy, providing objective responses ranging from 83 to 97% in humans and felines, respectively (8, 10, 26). ECT effectiveness is more pronounced in low-stage tumors; however, it can also provide clinical benefits for advanced lesions (8, 27).

Surgery is considered a first-line and effective local therapeutic modality that offers a greater chance of local cure, mainly for lesions located in the pinna of the ear, eyelid, and invasive lesions of the nasal planum (2). Lana et al. (28) evaluated different modalities of local treatment (surgery, radiotherapy, and cryosurgery) in 61 felines with SCC of the nasal planum and pina; surgery provided the longest disease-free period of 594 days. However, no studies have compared ECT with surgery, radiotherapy, or cryosurgery in cats with SCC. It is important to highlight that regardless of the treatment chosen, SCC treatment success depends on the clinical stage, tumor invasiveness, and location of the lesions (8).

Complete response was achieved in 96% (23/24) and PR in 4% (1/24) of the cats included in this study. The obtained CR rate was proportionally similar to the CR rate of 81.8% obtained by Tozon et al. (22), who prospectively evaluated 11 cats with superficial SCC treated with ECT. Similarly, Spugnini et al. (27) obtained a CR rate of 80.7% in cats with periocular SCC and advanced SCC of the head. Simčič et al. (8) obtained a CR rate of 65% in 61 retrospectively enrolled cats. However, comparisons must be made with caution, given the differences in sample sizes between the studies.

Local recurrence occurred in 13 of the 23 patients who achieved CR, resulting in a recurrence rate (RR) of 54%. This can be considered high when compared with other similar studies in which the RR ranged from 18 to 22% (8, 11). However, the heterogeneity between tumor location sites and tumor staging between different feline patients in these studies may have influenced the results, impairing a reliable comparison.

All PNI-positive cats developed local recurrence after CR (8/8), whereas only 31% (5/16) of the PNI-negative cats relapsed. Thus, local recurrence was significantly associated with PNI (*p* = 0.03). There is no information in the current literature regarding the occurrence of PNI in feline SCC. These preliminary results are the first published data on this issue and its correlation with local recurrence. In humans with cutaneous and oral SCC, the PNI is strongly associated with higher rates of local recurrence (14, 15, 29), corroborating the preliminary results of this study.

Despite the high recurrence rate, all PNI-positive cats achieved a satisfactory initial response (100% CR). It is difficult to discuss the causes because the mechanisms leading to PNI in feline SCC remain unknown. However, two hypotheses can be suggested: (1) a favorable initial response may be due to the high efficacy of ECT on tumor cells, regardless of the degree of malignancy; and (2) PNI may be an initially mild process; however, a progressive and silent dissemination pathway may cause persistence of microscopic disease, culminating in later local recurrence.

A higher number of ECT sessions (> 2 sessions) was necessary in half of the PNI-positive cats before CR achievement compared to the PNI-negative cats, which underwent one to two sessions. This suggests that PNI may play a role in the resistance and malignant mechanisms of SCC. Therefore, a greater number of ECT sessions can be recommended in PNI-positive cats, as well as close monitoring, even after achieving CR.

Although 83% of the SCC lesions (25/30) were from cutaneous tissues, three felines with oral and intranasal involvement were also included (Table 3). These patients developed SCC primarily in the oral cavity (patient #12; gingiva of left mandible, surrounding the caudal molar), and in the nasal cavity (patient #16). The other patient presented with concomitant intranasal and nasal planum lesions (patient #15), making it impossible to determine whether the intranasal disease was the result of cutaneous SCC progression or whether it was primary. Tozon et al. (22) did not find an association between the location of the lesion (ear or nasal planum) and the response to ECT, except in two patients who had cutaneous SCC and developed progressive disease in the nasal and oral cavities. In the present study, only one patient (patient #2; PNI-positive group; Table 2) presented with a similar course of the disease, with an initial lesion on the upper lip, which recurred and progressed to the nasal and oral cavities.

It is important to highlight that oral SCC is considered the most common oral cavity tumor in cats, presenting an overall worse prognosis, with lower survival rates and poorer response to established treatments than cutaneous SCC (22, 30). In turn, intranasal SCC represents approximately 10% of all intranasal tumors in cats, has locally invasive behavior, and as it requires a more complex surgical approach, effective local control becomes even more difficult than in cutaneous SCC (31). Similar to cats, oral SCC in humans is more aggressive than the cutaneous form. Several studies have investigated the occurrence of PNI in human oral SCC, revealing an incidence of up to 80% in these patients and an associated worse prognosis (19, 32, 33).

Despite the more aggressive nature and poorer response that is typical to oral SCC, the patient who developed a primary SCC in the oral cavity (patient #12—PNI-negative group), achieved a CR after only one ECT session. This favorable response can be due the early tumor stage at diagnosis, with tumor dimensions of approximately 2–2.5 cm (T2 stage) (34). In general, it is well-established that early stage SCC has a better response to ECT as well as a more favorable outcome, and this may have contributed to the good response (8, 9). In contrast, the another patient (patient #2—PNI-positive group) developed recurrence of a primary cutaneous SCC with progression into the oral and nasal cavity, which was treated with ECT but achieved a partial remission. However, these results are still preliminary and cannot confirm that that PNI-negative cats with oral SCC will respond better to ECT than PNI-positive cats particularly as the stage of disease for patients #12 (PNI-negative) and #2 (PNI-positive) were quite different.

It is important to highlight that three (12.5%) patients developed multiple lesions in different areas of the head (Table 2). All three patients were PNI-negative and only one did not recur. Furthermore, of the five PNI-negative cats with recurrence, two (40%) had lesions at multiple sites. SCC development in multiple locations on the head is relatively common, considering that it is a region that is more exposed to sunlight with less fur coverage, leading to carcinogenesis concomitantly in different exposed locations. Although there are no data in the current literature evaluating the number of lesions as a prognostic factor for SCC, treatment may become more complex as the number of lesions increases, leading to a higher risk of treatment failure and local recurrence.

Treatment safety was evaluated according to the local TOX (8, 24). Most patients were classified as having TOX > 2 (67%), with TOX 3 being the most prevalent score (56%). Sixty eight percent of the PNI-negative cats were classified as TOX > 2; however, there was no significant association (*p* > 0.05) between TOX and the occurrence of PNI. Owing to the differences in the sample sizes of the PNI groups, this result is still preliminary and may not be decisive. According to Simčič et al. (8), 49% of cats had TOX > 2 (53% were classified as TOX 4) and were three times more likely to suffer disease recurrence. However, in the present study, no statistically significant differences were observed between these variables (*p* > 0.05).

In addition to local recurrence, human SCC studies have shown a significant correlation between the presence of PNI, the occurrence of metastases, and shorter survival times (14, 15, 29). Unfortunately, given the retrospective nature of the present study, it was not possible to obtain survival data for all patients because they were lost to long-term follow-up. This made it impossible to obtain complete data on disease-free interval, survival time, and cause of death.

Tumor stage T (5) is defined by the depth of tumor invasion and tumor size, and the success of local treatment, whether by surgery, radiotherapy, cryosurgery, or ECT, is directly affected by these factors (2). As a relevant prognostic factor in feline SCC, the correlation between T stage and the presence of PNI is of fundamental importance in this study. However, it was not possible to classify all patients according to staging because of the lack of recorded information on tumor dimensions for some patients at the time of diagnosis and during treatment.

Despite these limitations, this study pioneered the evaluation of the PNI in feline SCC. The preliminary results suggest that the PNI may be a predictive factor for local recurrence in cats with SCC, highlighting its role as a potential prognostic marker, as already confirmed in humans. Along with these results, this study also describes the histopathological evaluation of PNI, which is of great value in encouraging the veterinary pathology community to examine this feature more often. Finally, these results may pave the way for new studies to be conducted prospectively with a larger sample size and considering different survival data to more assertively determine the prognostic value of PNI in feline SCC.

## Data availability statement

The original contributions presented in the study are included in the article/supplementary material; further inquiries can be directed to the corresponding author.

## Ethics statement

The animal studies were approved by National Council for the Control of Animal Experimentation (CONCEA) and was approved by the Animal Experimentation Ethics Committee of the Universidade Estadual Paulista “Júlio de Mesquita Filho” (UNESP/FCAV), Jaboticabal/SP (protocol no. 9849/2023). The studies were conducted in accordance with the local legislation and institutional requirements. Written informed consent was not obtained from the owners for the participation of their animals in this study because it was a retrospective study and consisted in medical records evaluation only.

## Author contributions

FS: Writing – original draft, Investigation, Data curation, Conceptualization. LL: Writing – review & editing, Writing – original draft, Methodology, Investigation, Formal analysis, Data curation, Conceptualization. MRo: Writing – review & editing, Visualization. KO: Writing – review & editing, Methodology, Investigation, Data curation. MRa: Writing – review & editing, Validation, Supervision, Project administration, Methodology, Conceptualization. AN: Writing – review & editing, Validation, Supervision, Project administration, Formal analysis, Conceptualization.
